# Estimating the Seroprevalence of Scrub Typhus in Nepal

**DOI:** 10.3390/pathogens13090736

**Published:** 2024-08-29

**Authors:** Piyada Linsuwanon, Nutthanun Auysawasdi, Chien-Chung Chao, Wuttikon Rodkvamtook, Binob Shrestha, Samita Bajracharya, Jasmin Shrestha, Sirima Wongwairot, Chawin Limsuwan, Erica Lindroth, Alyssa Mann, Silas Davidson, Elizabeth Wanja, Sanjaya Kumar Shrestha

**Affiliations:** 1 Department of Entomology, Walter Reed Army Institute of Research-Armed Forces Research Institute of Medical Science (AFRIMS), Bangkok 10330, Thailand; nutthanuna.ca@afrims.org (N.A.); sirimaw.ca@afrims.org (S.W.); chawinl.ca@afrims.org (C.L.); erica.lindroth.mil@afrims.org (E.L.); alyssa.mann.mil@afrims.org (A.M.); silas.a.davidson.mil@mail.mi (S.D.); elizabeth.w.wanja.mil@mail.mil (E.W.); 2 Naval Medical Research Command, Silver Spring, MD 10400, USA; ch3chao@gmail.com; 3 Analysis Division, Royal Thai Army-Armed Forces Research Institute of Medical Science, Bangkok 10400, Thailand; rwuttikon@gmail.com; 4 Walter Reed/AFRIMS Research Unit Nepal, Kathmandu 44600, Nepal; binobs.ca@afrims.org (B.S.); samitab.ca@afrims.org (S.B.); jasmins.ca@afrims.org (J.S.); shresthask.ca@afrims.org (S.K.S.)

**Keywords:** scrub typhus, Nepal, serosurveillance, recombinant protein ELISA, acute febrile illness, *Orientia tsutsugamushi*

## Abstract

Prior to the devastating earthquake in Nepal in 2015, scrub typhus was not recognized as a highly endemic disease in the country. This contrasted with neighboring India, where scrub typhus is endemic and there have been sporadic outbreaks of severe forms. This discrepancy underscores the limitations in our comprehensive understanding of the scrub typhus epidemiological patterns in Nepal, especially before 2015. To better understand the dynamic and current status of scrub typhus, this study investigated its prevalence among patients with acute febrile illness in two hospitals located in Pokhara city, Kaski district and Bharatpur city, Chitwan district during 2009–2010. Our findings revealed that 31.5% (239 of 759 patients) of the cases were positives for scrub typhus based on serological and pathogen detection assays. These results provide crucial insights into the pre-earthquake endemicity of scrub typhus in Nepal, implying its long-standing presence in the region prior to the significant environmental transformations caused by the 2015 earthquake. This study also emphasizes the need for heightened awareness and improved diagnostic capabilities to effectively manage and control scrub typhus, which remains a significant public health concern in Nepal.

## 1. Introduction

Scrub typhus is a mite-borne disease predominantly found in the Asia-Pacific region, specifically within the area known as the Tsutsugamushi Triangle. In recent years, scrub typhus has begun to emerge in previously unrecognized regions including North and South America and Africa, indicating the potential for a global distribution [1,2,3,4,5]. The causative agent of scrub typhus is a Gram-negative, rod-shaped bacterium from the *Orientia* genus, which is distinct from the *Rickettsia* genus by the absence of lipopolysaccharide in its cell wall. Currently, one species and two potentially new species are recognized within the genus *Orientia*: *Orientia tsutsugamushi* (*O. tsutsugamushi*), *Candidatus* O. chuto, and *Ca.* O. chiloensis [6]. The species and genotypes of *Orientia* vary by geographic location due to the diversity of the pathogen and its arthropod vectors. Transmission of *Orientia* bacteria occurs through the infectious bite of trombiculid larval mites, which represent the only parasitic stage of their life cycle capable of transmitting the pathogen to hosts. Among the 28 genera of trombiculid mites, *Leptotrombidium* is the most predominant genus identified in the endemic areas, and these serve as competent vectors in pathogen transmission [7].

Scrub typhus is a life-threatening disease that can lead to severe complications and fatalities due to multi-organ failure if treated improperly [8]. Despite its severity, the disease has been neglected in many parts of the world, often due to a lack of awareness, frequent misdiagnoses with other indistinguishable tropical diseases, and limited resources for detection and treatment [9]. A major earthquake occurred in Nepal in 2015, reportedly measuring a magnitude of 7.9 on the Richter scale, which led to widespread destruction, loss of life, and displacement of communities. The natural disaster not only affected infrastructure, healthcare systems, and the living conditions of people [10,11] but also altered the ecological balance, potentially increasing the habitats suitable for arthropod vectors and small rodents, as well as bringing ectoparasitic pests they may enter into close proximity with humans. Despite the Nepalese Government being notified of scrub typhus as a reportable disease, the disruption of many sectors caused by the earthquake and its aftermaths significantly hindered disease surveillance and reporting. This likely led to an underestimation of scrub typhus cases at the time, complicating efforts to understand and address the disease during a period when scrub typhus transmission may have been intensifying. Consequently, there was an unexpected surge in scrub-typhus-related fatalities, with scrub typhus outbreaks occurring in many regions of the country. During 2015–2017, the scrub-typhus-related fatality rates reached at least 4.6% [12,13]. This highlights the critical need for comprehensive disease and vector surveillance, coupled with a prompt medical response, particularly in post-disaster settings where the usual health infrastructure and reporting mechanisms may be compromised. Additionally, an adequate understanding of the local population’s serological background of scrub typhus is crucial for the development of appropriate diagnostic determination for sample positivity, especially when the diagnosis relies only on single acute serum samples. Improved diagnostics may reduce the mortality rate of scrub typhus through timely, proper antibiotic treatment.

To enhance the effectiveness of management and control strategies for scrub typhus diagnosis in Nepal, this study developed appropriate criteria for scrub typhus diagnosis through a comprehensive analysis of the infection’s prevalence, achieved by employing both serological and molecular methods. By focusing on the provinces that were affected by the 2015 earthquake and the period before the event, which present gaps in our knowledge, we also provide comprehensive information on the epidemiological dynamic of scrub typhus in Nepal. This endeavor is expected to establish a solid foundation for future epidemiological studies, significantly contributing to the refinement and improvement of diagnostic criteria for scrub typhus.

## 2. Materials and Methods

### 2.1. Study Sites, Patient Samples, and Ethical Approval

Nepal is geographically divided into three distinct regions running from the north to the south: the Mountain, Hill, and Terai regions. The Mountain region or the Himalayas is the northernmost region of Nepal, which is characterized by high peaks, including Mount Everest, deep valleys, and a sparse population due to its rugged terrain. The region is cold, and many areas remain snow-covered throughout the year. The Hill region is located to the south of the Mountain region, encompassing the Mahabharat Range and the lower Chure Hills. It has a temperate climate and is more populated than the Mountain region. The Terai region is the southernmost region of Nepal and is characterized by flat plains bordering India. The Terai region has a warm, subtropical climate and is the most populated and agriculturally productive area of the country.

**Figure 1:**
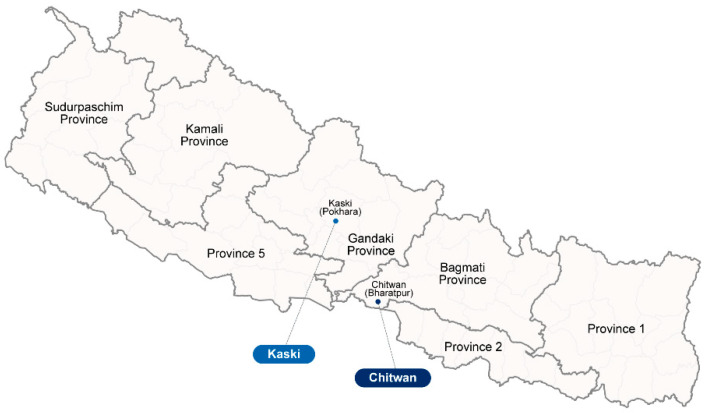
Map of Nepal indicating the two locations where acute febrile illness patients and healthy volunteers were recruited for this study.

In our cross-sectional descriptive study, patients with acute febrile illness (AFI) who presented to the hospitals located in Pokhara city, Kaski district and Bharatpur city, Chitwan district were enrolled in the study (Figure 1). These locations were chosen as representatives of the Hill and Terai regions, respectively. The enrollment covered the period between September 2009 and November 2010. Patients included in this study were those with AFI who showed no evidence of a primary focus of infection. Both in and outpatients aged greater than or equal to 2 years old who met the inclusion criteria described in Table S1 were included in this study. All participants and consignees were informed about the potential use of their residue samples for future research. Ethical approval of this research was granted from both the Ethical Review Board of the Nepal Health Research Council (NHRC Reg. No. 207/2018) and the Human Subjects Protection Branch of the Walter Reed Army Institute of Research (WRAIR protocol number 2490).

Paired serum samples collected during the first hospital visit (acute-phase samples) and at 21 days post initial sampling (convalescent-phase samples) were collected from AFI patients and used for serological and pathogen analysis. In total, 759 AFI patients participated in this study, of whom 449 patients were from Bharatpur Hospital in Bharatpur city, Chitwan and 310 patients were from Western Regional Hospital in Pokhara city, Kaski. Given that this sample set was initially screened for a range of vector-borne pathogens significant to public health, we incorporated partial pathogen screening results into our study for comparative analysis. This included the results of *O. tsutsugamushi* detection using a nested polymerase chain reaction (PCR) assay targeting the 56 kDa type-specific antigen gene (56*tsa*) encoded for the dominant protein membrane [14], genus-specific *Rickettsia* using PCR for the 17 kDa type-specific antigen gene (17*tsa*) [15], and dengue virus infection using an in-house real-time reverse transcription PCR assay.

### 2.2. Enzyme-Linked Immunosorbent Assay for O. tsutsugamushi-Specific Antibodies

The specific serology of AFI patients against *O. tsutsugamushi* was evaluated using a recombinant-protein-based enzyme-linked immunosorbent assay (ELISA) following a previously established protocol [16,17]. Recombinant protein antigens developed from the most abundant and immunodominant 56 kDa protein of *O. tsutsugamushi* strain Karp and TA763 (r56C1), Kato (r56kKt), and Gilliam (r56Gm) were expressed using an *Escherichia coli* expression system. These recombinant proteins were purified using the high-performance liquid chromatography method [18]. The mixture, containing equal concentrations of each antigen protein, was prepared by combining them in a ratio of 1:1:1 and then diluted in 1× phosphate-buffered saline (1× PBS) to achieve the final concentration of 0.1 µg total proteins/100 µL 1× PBS for ELISA plate coating. The antigen-coated plates were stored at 4 °C for two nights before being washed three times with 1× PBS and blocked with 10% *w*/*v* skim milk in 1× PBS to remove excess recombinant protein antigens. Serum samples were diluted to 1:100 in 1% skim milk/1× PBS and were transferred to the antigen-coated plates and incubated for 1 h. Following the incubation step, the plates were washed three times with 1× PBS/0.5% Tween-20 solution. The enzymatic reaction between horse-radish peroxidase-conjugated anti-human IgM or IgG antibodies at a concentration of 1:14,000 and tetramethylbenzidine substrate was determined after inoculation for 30 min by measuring the optical density (OD) of the sample at 405 nm (OD_405_) and reference OD at 650 nm (OD_650_) using a Thermo Scientific™ Multiskan™ GO microplate reader (Thermo Fisher Scientific, Waltham, MA, USA). The net OD of each sample for data analysis was calculated from the OD_405_ by subtracting the OD_650_. Positive samples at a 1:100 dilution were then subjected to two-fold serial dilution starting from 1:100 and running to 1:12,800, to determine the endpoint titer.

To ensure the quality control of our experiments, a panel of reference samples, which included purposive sets of serum samples from Thailand with positive and negative statuses confirmed by an immunofluorescent staining assay (IFA) and 56*tsa* PCR of *O. tsutsugamushi,* as well as healthy populations in Nepal, were also included in all ELISA batches. The positive control samples, which included serum samples that contained specific IgM and IgG at the IFA titers, ranged from 1:100 to 1:3200 for both types. For consistency across different ELISA experiments, the average OD of the same sets of positive and negative control samples was required to fall within the range of one standard deviation (SD).

## 3. Data Interpretation and Analysis

Recently, a variety of methods has been introduced to determine the optimal cutoff for scrub typhus ELISA. However, universally accepted standard criteria for interpreting the data remain unavailable. In our study, we analyzed and compared the results obtained from two different cutoff calculation methodologies. The first method for cutoff determination was an average optical density cutoff plus SD at the 99% confidence interval (OD + SD (99% CI)) [19]. This cutoff, referred to as the ‘endemic cutoff’, relies on the serological background of the Nepalese population in the study areas. It has been considered a reliable algorithm for accurately diagnosing scrub typhus, especially in endemic areas where the populace might exhibit an elevated serological background. The high background could potentially lead to a higher likelihood of positive results and a higher proportion of seropositive cases, especially when only a single acute-phase serum sample is available for the diagnostic test. The use of an endemic OD cutoff also yields results comparable to those obtained through IFA [17]. The second method was a determination of the optimal OD cutoff as the average OD of true negative samples plus three times its standard deviation. This method has demonstrated accuracy and power in differentiating the true positive cases and has been used for the interpretation of commercial ELISA test results for scrub typhus [20,21,22]. To determine the cutoff values, serum samples from healthy volunteers were analyzed using an in-house recombinant-protein-based ELISA as described in prior sections. The sample sets included 511 samples from Chitwan and 266 samples from Kaski. The samples were collected during 2017 to 2018, supported by the Nepal Red Cross Society. Patients with active scrub typhus infection were identified based on one of the following criteria as shown in Table 1. (a) acute IgM or IgG titer ≥ 1:400, (b) four-fold increase in IgM or IgG titers between the acute and convalescent phases, or (c) positive results for 56*tsa* PCR.

### Statistical Analysis

Statistical analysis was conducted using Prism-GraphPad Software (version 9). The analysis included frequency distributions, percentages, and the Chi-square test. A *p*-value of less than 0.05 was considered statistically significant. Odds ratios with 95% CI were used to assess the impact of various demographic factors on scrub typhus. This comprehensive approach provided us with confidence in the assessment of the impacts of various demographic factors on scrub typhus.

## 4. Results

### 4.1. ELISA Diagnostic Cutoff

During the study period, a total of 759 AFI patients were evaluated for the presence of specific antibodies against the key genogroups of *O. tsutsugamushi*. Among scrub-typhus-positive cases, 54.5% (414 cases) were male, resulting in a male-to-female ratio of 1.2:1. There was no difference in gender between different age groups (*p*-value = 0.7116). Approximately 77.2% of the enrolled cases were under 30 years of age, with the vast majority being children and adolescents aged 11 to 20 years old. The median age of enrolled patients was 19 ± 14.6 years old, with an interquartile range for the first and third quartiles (IQR1-3) of 11–30 years old. The median day of symptom onset before acute-phase sample collection was 5 ± 2.9 days (IQR1-3: 3–7 days), while the median number of days before convalescent sample collection was 22 ± 17.1 days (IQR1-3: 21–25 days).

**Figure 2:**
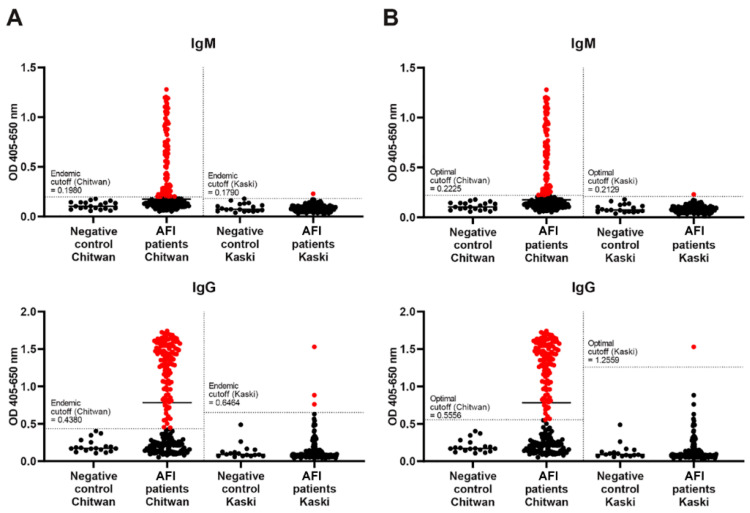
Comparison of diagnostic cutoffs for scrub typhus using two different methods: (**A**) endemic cutoff value and (**B**) optimal optical density (OD) cutoff value. Data points marked with red dots represent seropositive results, indicating the samples with OD higher than the respective cutoff values.

When comparing the prevalence of scrub typhus using serological analysis with two different cutoff calculation methods, the endemic cutoff method yielded a scrub typhus prevalence at 1.2 times higher than the optimal OD cutoff method (228 vs. 193 cases) (Figure 2). However, the difference between the two methods was not statistically significant. Therefore, our recent work employed the endemic cutoff method for data analysis and interpretation. We proposed the cutoff value for scrub typhus IgM at 0.51 and IgG at 0.42 for Bharatpur city, Chitwan district, while the cutoff value for IgM was 0.73 and IgG was 0.55 for Pokhara, Kaski (Figure 2). These cutoff values were determined based on our investigation of representative sets of serum samples from healthy volunteers in the respective locations.

### 4.2. Demographic Variables of Scrub Typhus Patients

**Figure 3:**
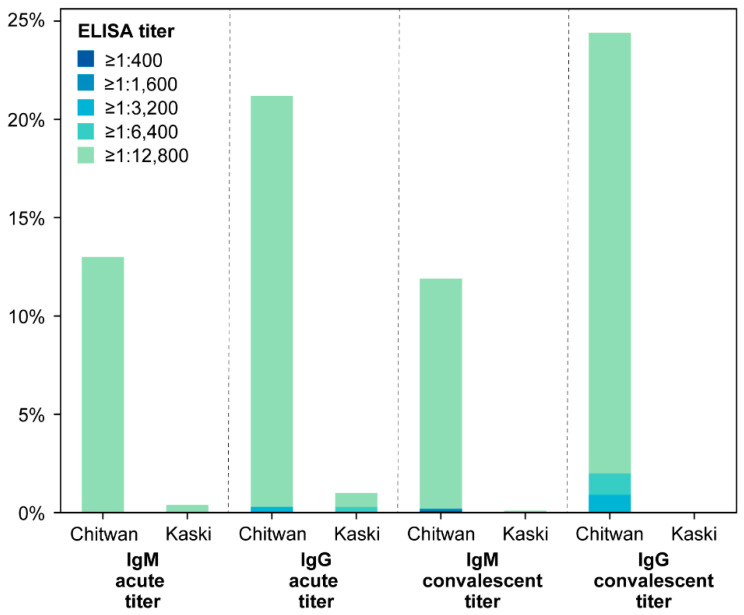
Distribution of antibody titer in Chitwan and Kaski.

When serological and molecular analyses were combined, 10% (239 cases) of the AFI patients were identified as having a scrub-typhus-positive status according to the diagnostic criteria (Table 1). Of these scrub-typhus-positive cases, 10% were confirmed as having scrub typhus infection based on either ELISA or PCR assay, while 21.5% were considered probable cases due to the presence of IgG or IgM ≥ 1:400 in single sera (Table 1). A total of 25.5% (61 cases) demonstrated a four-fold increase in either IgM or IgG titers. In the acute phase, 4.4% (10 cases) of scrub typhus cases showed early rising IgM titers, followed by an increase in either IgM or IgG. A total of 46.1% (105 cases) exhibited a singular elevated IgM titer surpassing 1:400, with 105 of these cases reaching an acute IgM titer ≥ 1:12,800 (Figure 3). The IgG ELISA results also highlighted variations in infection status among AFI patients. A total of 45.6% (104 of 228 cases) presented IgG titers over 1:400, in the absence of elevated IgM (Table S2). Given the strain-specific nature of antibodies against *O. tsutsugamushi*, the observed high IgG titers during the acute phase suggest either recurrent exposure to homologous strains or a predominant circulation of specific *O. tsutsugamushi* strains within the study areas. Additionally, it was observed that none of the patients were in the recovery phase or had recovered from scrub typhus from a previous episode, as there was no evidence of IgG titers below 1:400 without a corresponding four-fold increase in IgM titers.

**Figure 4:**
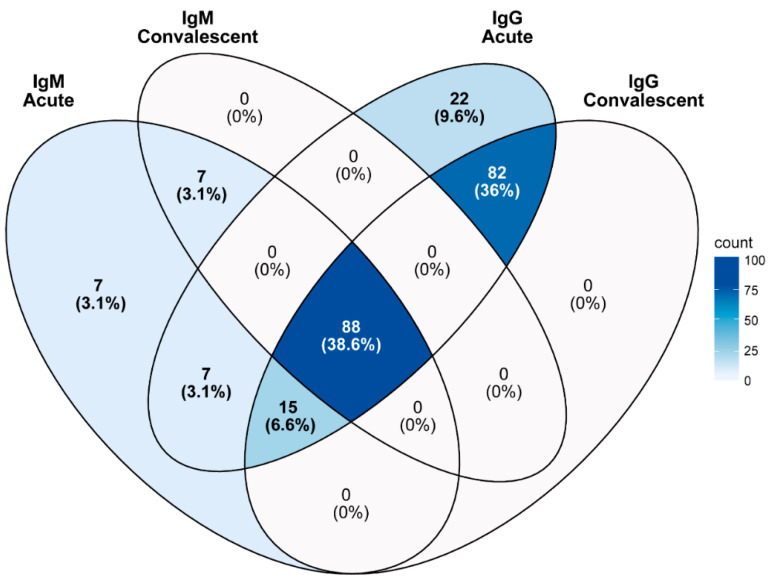
Venn diagram of IgM and IgG antibody distribution in the acute and convalescent phases in Nepal (n = 228).

Table 2 shows associations of demographic characteristics of scrub typhus patients in Nepal. Female patients exhibited a slightly higher seropositive status than males, with a ratio of 1.1 females to 1 male. This difference was statistically significant (odds ratio = 1.495, *p*-value = 0.0128, 95% CI = 1.099–2.034). However, when separated by study locations, a relatively similar ratio of males and females with active scrub typhus infection was observed (Table 3). The age group of 11 to 20 years had the highest rate of scrub typhus positivity, at 9.6%, followed by the 21 to 30 years age group at 6.6%, and the 31 to 40 years age group at 5.5% (Table 2). These findings suggest that both gender and age have significant correlations with scrub typhus positivity. The data also suggest a cumulative exposure to the pathogen over time, which could be due to outdoor activities or occupational exposure, as indicated by the percentage of IgG-positive results in the acute phase (9.6% (22 cases)), pointing towards secondary or recurrent infections (Table 4 and Figure 4).

### 4.3. Co-Infection with Other Pathogens

Patients with confirmed or suspected scrub typhus had a median duration of symptom onset before admission of 6 ± 3.1 days (IQR1-3: 4.0–8.7 days) and convalescence of 22 ± 19.4 days (IQR1-3: 21–25 days). The clinical manifestations of scrub typhus were prominently observed in our study, with fever (99.1%), a headache (84.6%), malaise (82%), anorexia (80.3%), fatigue (78.5%), muscle aches (78.1%), and chills (72.8%) being the predominant symptoms, as reported in over 70% of confirmed cases (Table 3). While the incidence of fever, headache, and malaise was slightly higher in IgM- or PCR-positive cases as compared to IgG-positive cases, this difference did not achieve statistical significance.

A total of 0.5% (4 of 759) of AFI patients were found to be infected with a *Rickettsia* pathogen, while 5.9% (45 cases) tested positive for dengue virus infection. Co-positivity of *O. tsutsugamushi* and dengue virus in the patients was identified in 2.6% (20 cases), always among AFI patients from Bharatpur, Chitwan, one of the endemic areas of dengue virus in Nepal. These patients tended to have a shorter duration of symptom onset before admission (median 5 ± 2.8 days, IQR1-3: 3.0–6.5 days) compared to the patients with sole infection (median 6 ± 3.1 days, IQR1-3: 4–9 days). This observation aligns with the complex clinical presentations seen in the patients with co-infection, and we propose that the more pronounced symptoms could be the driving factor behind these patients seeking medical care and subsequently being admitted to hospital. The occurrence of co-infections highlights the diagnostic challenges of febrile illnesses in Nepal and underscores the need for a comprehensive diagnostic strategy.

### 4.4. Population Background for Scrub Typhus Infection

Due to the unavailability of serum samples from the healthy population prior to 2015, we decided to investigate the population background of Nepalese blood donors concerning scrub typhus infection using serum samples collected between 2017 and 2018. The results indicated that 16.4% (84 of 511) of the healthy population living in Bharatpur, Chitwan district demonstrated evidence of past exposure to the scrub typhus pathogen, based on the presence of specific IgG in the sera, suggesting a potential undetected prevalence in the community. The seroprevalence of the Chitwan population was 1.2 times higher when compared to the data obtained from Kaski (20.3%; 54 of 266).

### 4.5. Geography, Seasonality, and Dynamics of Scrub Typhus in Nepal

**Figure 5:**
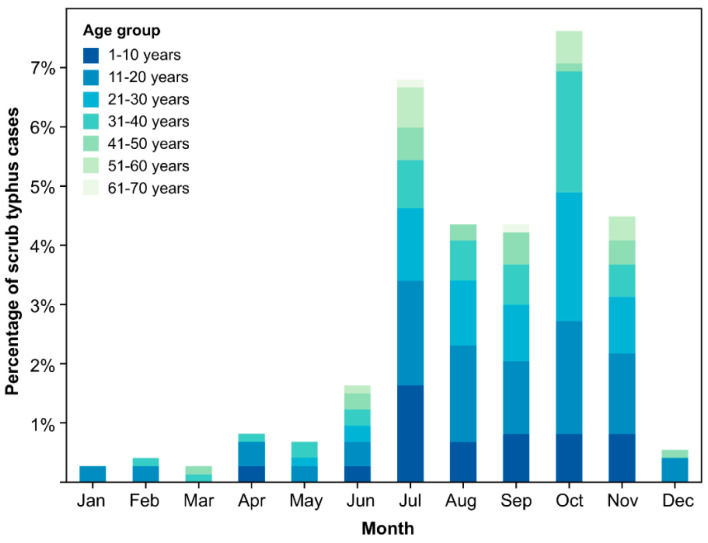
Occurrence and temporal distribution of confirmed scrub typhus cases by month, indicating the highest incidence of scrub typhus in the month of October in Chitwan.

A significantly higher prevalence of scrub typhus infection was observed in Chitwan compared to Kaski, suggesting that geographical or environmental factors in Chitwan make it more conducive to scrub typhus transmission (48.8% (219 of 449 cases) in Chitwan vs. 6.5% (20 of 310 cases) in Kaski). In Chitwan, the monthly infection prevalence ranged from 0.2% to 12%, with a noticeable increase in active cases from July to November. This timeframe aligns with the monsoon season of Nepal, during which moist and humid conditions may be favorable for the proliferation of chigger vectors. Additionally, the monsoon season might alter human outdoor activities, potentially increasing exposure to areas where chiggers are abundant. This observation was validated by an analysis of seasonal variation, where cases of scrub typhus in Chitwan were significantly associated with the summer season (*p*-value = 0.0002) (Figure 5).

## 5. Discussion

To date, multiple research groups have conducted comparative studies to evaluate the efficiency of both in-house and commercial ELISA methods. Our team’s preliminary analysis comparing ELISA to the gold-standard IFA method reveals that ELISA is comparable to the gold-standard IFA method in terms of sensitivity (66.7%; range from 35.9% to 91.4%), specificity (100%; range from 21.7% to 100%), and accuracy (75%; range from 48.1% to 97.9%) (unpublished data). Given that IFA method is subjective and requires both specialized equipment and skilled personnel for the evaluation and verification of microscopic results, ELISA has been considered as a replacement reference method, especially in resource-limited areas [17,23,24,25]. Our serological analysis suggests that scrub typhus is more common compared to other rickettsioses and presents as one of the main causes of AFI in Nepal. We also observed that distinct geographic regions displayed varying risks of scrub typhus exposure. In Chitwan district, we observed a remarkably high infection prevalence, with the rate reaching as high as 48.8%. The scrub typhus activity in Chitwan also exhibited a distinct seasonal pattern when compared to Kaski and closely resembled the distribution pattern reported by previous studies [26]. This seasonal activity of scrub typhus underscores the importance of refining public health interventions to align with these trends. We also observed the decline in the infection prevalence of scrub typhus in recent years. For instance, during the sample collection period from 2015 to 2017, the infection prevalence was recorded to be as high as 61% among acute febrile illness patients [13]. Subsequent surveillance data from the most recent years indicated a further decrease, with infection rates ranging from 13% to 22.6% [27,28,29]. This decline in infection prevalence may be attributed to several factors, including heightened awareness of scrub typhus within the local community and improved access to prompt antibiotic treatment for suspected cases. Targeted public health interventions coupled with education campaigns aimed at raising awareness about the disease and its vector may contribute to this positive trend.

The findings of our study should be interpreted with consideration of certain limitations. First, the reliance on PCR detection for determining the prevalence of *Rickettsia* and dengue virus might have contributed to their observed lower prevalence rates in comparison to scrub typhus. PCR-based methods, while highly specific, might not capture all infections, especially if the viral or bacterial load is below the detection threshold or if there are variations in the targeted genetic sequences. Second, we observed a variation in ELISA results across different batches, despite the consistent inclusion of the same set of positive and negative control samples in every experiment. To address this, we established an acceptable range based on one standard deviation for these control samples and utilized this criterion for data analysis. As a result, 859 samples that fell outside this range or potentially affected the overall findings were excluded from this study.

Our present study addresses a critical knowledge gap regarding the threat of scrub typhus in Nepal and highlights the persistently high prevalence of scrub typhus among the Nepalese population, even before the occurrence of the massive earthquake event. This emphasizes the status of scrub typhus as a neglected but significant public health concern in Nepal. Knowledge gained from our study should help the authorities in implementing appropriate therapeutic interventions and establishing preventive measures to reduce morbidity and mortality associated with scrub typhus. Additionally, our findings underscore the importance of continuous surveillance and the need for heightened awareness among healthcare professionals regarding scrub typhus. Scrub typhus should be considered in the differential diagnosis of fever with an unknown origin. Further studies are warranted to understand the full extent of the disease prevalence and its potential impact on public health in Nepal.

## Figures and Tables

**Table 1:** Diagnostic criteria for scrub typhus.

Diagnosis	Serology Result	% Positivity
Scrub typhus	Suspected cases: single titer of IgG or IgM or both ≥400	21.5% (163)
	Confirmed cases: 4-fold increase in IgG or IgM titer in paired samples	8.0% (61)
	Confirmed cases: 56*tsa* PCR positive only	1.4% (11)
	Confirmed cases: Positive in both serology and PCR	0.6% (4)
Unknown	Negative by all criteria	68.5% (520)

**Table 2:** Demographic characteristics of acute febrile illness patients who tested positive for scrub typhus infection.

Variable	Febrile Cases (n = 759)	Scrub Typhus Cases						*p*-Value	Odds Ratio	95% Confidence Interval
		Total Cases (n = 759) ^a^		Female (n = 345) ^a^		Male (n = 414) ^a^				
		No.	%	No.	%	No.	%			
**Scrub Typhus Case**										
Positive	239	239	(31.5%)	125	(36.2%)	114	(27.5%)	0.013 *	1.495	1.099–2.034
Negative	520	520	(68.5%)	220	(63.8%)	300	(72.5%)		1	
**Age Group ^b^**										
1–10 years	177	39	(5.1%)	17	(4.9%)	22	(5.3%)	1	0.773	0.045–13.268
11–20	241	73	(9.6%)	33	(9.6%)	40	(9.7%)	1	0.825	0.049–13.702
21–30	168	50	(6.6%)	27	(7.8%)	23	(5.6%)	1	1.174	0.069–19.835
31–40	91	42	(5.5%)	30	(8.7%)	12	(2.9%)	1	2.5	0.144–43.287
41–50	41	18	(2.4%)	10	(2.9%)	8	(1.9%)	1	1.25	0.067–23.261
51–60	26	13	(1.7%)	6	(1.7%)	7	(1.7%)	1	0.857	0.044–16.850
61–70	11	2	(0.3%)	1	(0.3%)	1	(0.2%)	1	1	0.020–50.400
71 and above	4	2	(0.3%)	1	(0.3%)	1	(0.2%)		1	
**Location ^b^**										
Chitwan	449	219	(28.9%)	116	(33.6%)	103	(24.9%)	0.653	1.376	0.548–3.454
Kaski	310	20	(2.6%)	9	(2.6%)	11	(2.7%)		1	
**Seasonal Variation ^b^**										
**2009–2010**										
January	20	3	(0.4%)	1	(0.3%)	2	(0.5%)		1	
February	21	3	(0.4%)	2	(0.6%)	1	(0.2%)	1	4	0.134–119.237
March	30	2	(0.3%)	0	(0%)	2	(0.5%)	1		
April	48	6	(0.8%)	1	(0.3%)	5	(1.2%)	1	4	0.016–10.017
May	57	5	(0.7%)	3	(0.9%)	2	(0.5%)	1	3	0.15–59.893
June	44	12	(1.6%)	7	(2%)	5	(1.2%)	0.897	2.8	0.196–40.059
July	134	50	(6.6%)	22	(6.4%)	28	(6.8%)	1	1.571	0.134–18.478
August	97	32	(4.2%)	14	(4.1%)	18	(4.3%)	1	1.556	0.128–18.951
September	82	32	(4.2%)	17	(4.9%)	15	(3.6%)	0.959	2.267	0.186–27.583
October	99	56	(7.4%)	34	(9.9%)	22	(5.3%)	0.736	3.091	0.264–36.169
November	77	34	(4.5%)	21	(6.1%)	13	(3.1%)	0.728	3.231	0.266–39.287
December	50	4	(0.5%)	3	(0.9%)	1	(0.2%)	0.741	6	0.221–162.541

Notes: * represents *p*-values < 0.05 that were considered significant when comparing females and males, “^a^” represents the number of tested cases, “^b^” represents the positive percentage of each group in relation to the total tested cases. The scrub-typhus-positive cases were determined based on the results of serology and PCR assays.

**Table 3:** Comparison of demographic variables and scrub typhus infection between Chitwan and Kaski populations.

Variable	Chitwan			Kaski			*p*-Value
	Febrile Cases (n = 449)	Scrub-Typhus-Positive Cases (n = 219)		Febrile Cases (n = 310)	Scrub-Typhus-Positive Cases (n = 20)		
**Sex**							
Male	247	103	(22.9%)	167	11	(3.5%)	<0.0001 *
Female	202	116	(25.8%)	143	9	(2.9%)	<0.0001 *
**Age Group**							
1–10 Years	98	38	(8.5%)	79	1	(0.3%)	<0.0001 *
11–20	137	62	(13.8%)	104	11	(3.5%)	<0.0001 *
21–30	102	46	(10.2%)	66	4	(1.3%)	<0.0001 *
31–40	64	41	(9.1%)	27	1	(0.3%)	<0.0001 *
41–50	25	17	(3.8%)	16	1	(0.3%)	0.0004 *
51–60	14	12	(2.7%)	12	1	(0.3%)	0.0004 *
61–70	6	2	(0.4%)	5	0	(0%)	0.5207
71 and above	3	1	(0.2%)	1	1	(0.3%)	1
**Seasonal Variation**							
**2009–2010**							
January	5	1	(0.2%)	15	2	(0.6%)	1
February	6	1	(0.2%)	15	2	(0.6%)	1
March	7	1	(0.2%)	23	1	(0.3%)	0.9540
April	19	5	(1.1%)	29	1	(0.3%)	0.0579
May	22	3	(0.7%)	35	2	(0.6%)	0.5834
June	18	11	(2.4%)	26	1	(0.3%)	0.0001 *
July	97	44	(9.8%)	37	6	(1.9%)	0.0035 *
August	60	32	(7.1%)	37	0		<0.0001 *
September	66	31	(6.9%)	16	1	(0.3%)	0.0067 *
October	81	54	(12%)	18	2	(0.6%)	0.0001 *
November	58	33	(7.3%)	19	1	(0.3%)	0.0002 *
December	10	3	(0.7%)	40	1	(0.3%)	0.0267 *

Note: * represents *p*-values < 0.05 that were considered significant when comparing Chitwan and Kaski.

**Table 4:** Comparison of scrub typhus suspected and confirmed patient characteristics and ELISA results in primary vs. secondary infections.

Patient Characteristic	Cases (n = 228)	Primary Infection (n = 102)	Secondary Infection (n = 126)	*p*-Value
Age, years (mean ± SD)	25 ± 15.2	23.1 ± 14.2	26.6 ± 15.8	0.0873
Sex	Female 53.5% (122)	Female 61.8% (63)	Male 53.2% (67)	0.0318 *
ONSET-ENRLD	6.3 ± 3.2	7.9 ± 3.1	5 ± 2.5	<0.0001 *
ENRLD-FU	29 ± 19.8	27.1 ± 15.6	30.5 ± 22.6	0.1985
ONSET-FU	35.3 ± 20.1	35 ± 16.4	35.5 ± 22.7	0.8400
Body temperature (°C)	39.2 ± 3.1	39.3 ± 1.3	39.1 ± 4	0.5720
Pulse /min	98.5 ± 11.8	99.3 ± 13	97.9 ± 10.7	0.3550
Bp (D)	98.2 ± 13.6	96.7 ± 13.2	99.5 ± 13.8	0.1146
Bp (S)	61.9 ± 10.3	61.1 ± 9.7	62.5 ± 10.8	0.2899
Respiratory rate/min	21 ± 3.2	21.7 ± 4.4	20.5 ± 1.7	0.0014 *
Fever	226 (99.1%)	102 (100%)	124 (98.4%)	0.3797
Headache	193 (84.6%)	89 (87.3%)	104 (82.5%)	0.5054
Malaise	187 (82%)	85 (83.3%)	102 (81%)	0.6931
Anorexia	183 (80.3%)	84 (82.4%)	99 (78.6%)	0.4384
Fatigue	179 (78.5%)	83 (81.4%)	96 (76.2%)	0.3396
Muscle Aches	178 (78.1%)	79 (77.5%)	99 (78.6%)	0.4333
Chills	166 (72.8%)	80 (78.4%)	86 (68.3%)	0.0665
Retro-orbital Pain	116 (50.9%)	57 (55.9%)	59 (46.8%)	0.1084
Joint Pain	105 (46.1%)	47 (46.1%)	58 (46%)	0.6385
Nausea	99 (43.4%)	43 (42.2%)	56 (44.4%)	0.7815
Cough	89 (39%)	40 (39.2%)	49 (38.9%)	0.9092
Vomiting	87 (38.2%)	41 (40.2%)	46 (36.5%)	0.3614
Abdominal cramps	76 (33.3%)	38 (37.3%)	38 (30.2%)	0.1833
Chest pain	52 (22.8%)	29 (28.4%)	23 (18.3%)	0.0530
Sore throat	52 (22.8%)	22 (21.6%)	30 (23.8%)	0.6843
Rhinorrhea	42 (18.4%)	19 (18.6%)	23 (18.3%)	0.7434
Diarrhea	17 (7.5%)	5 (4.9%)	12 (9.5%)	0.4180
Coryza	13 (5.7%)	6 (5.9%)	7 (5.6%)	0.6204
Conjunctivitis	12 (5.3%)	7 (6.9%)	5 (4%)	0.8415
Rash	12 (5.3%)	7 (6.9%)	5 (4%)	0.1156
Loss of consciousness	9 (3.9%)	1 (1%)	8 (6.3%)	0.0396 *
Wheezing	8 (3.5%)	7 (6.9%)	1 (0.8%)	0.0207 *
Shortness of breath	7 (3.1%)	4 (3.9%)	3 (2.4%)	0.3281
Seizures	6 (2.6%)	2 (2%)	4 (3.2%)	0.9237
Jaundice	4 (1.8%)	2 (2%)	2 (1.6%)	0.8769
Confusion	2 (0.9%)	0	2 (1.6%)	0.1954
Stiff neck	2 (0.9%)	1 (1%)	1 (0.8%)	0.3613
Eschar lesion	1 (0.4%)	0	1 (0.8%)	0.3748
Other	1 (0.4%)	0	1 (0.8%)	0.3765
Bleeding	0 (0%)	0	0	

Note: * represents *p*-values < 0.05 that were considered significant when comparing primary and secondary infection.

## Data Availability

The original contributions presented in the study are included in the article/Supplementary Material, further inquiries can be directed to the corresponding author.

## Supplementary Materials

The following supporting information can be downloaded at: https://www.mdpi.com/article/10.3390/pathogens13090736/s1, Table S1. Inclusion and exclusion criteria, Table S2. Detailed serological diagnostic results for scrub typhus infection.
