# Supplementary material for: Estimating the Seroprevalence of Scrub Typhus in Nepal
Source: Pathogens. 2024 Aug 29;13(9):736. doi: 10.3390/pathogens13090736 (PMC11435068; doi:10.3390/pathogens13090736)
Supplement: Supplementary file 1 [file pathogens-13-00736-s001.zip › pathogens-3153230-supplementary.pdf]

Supplement Tables

Table S1. Inclusion and exclusion criteria

	Category description	
**Criteria**	**Inclusion Criteria**	**Exclusion Criteria**
Age	Male or female ≥ 2 years old	Less than 2 years old
Fever duration	Fever (≥38°C (oral or rectal) or ≥37°C axillary) for at least 24 hours but less than 14 days, or documented history within 72 hours of presentation	Febrile for greater than 14 days
Consent	Consent form signed by subject or proxy	No signed consent form
Specific symptoms	No	Chief complaint limited to specific systems (respiratory, gastrointestinal) or identified focus of infection (otitis media, sinusitis, etc.)
Prior diagnosis	No	Febrile illness already diagnosed via lab tests
Unsuitable subject	No	Investigator judgement: not optimal for study participation (e.g., non-infectious source of fever)
Documented fever	Yes	No documented fever as defined above
HIV infection	No	Known HIV infection
Pregnancy	No	Currently pregnant
Follow-up	No	Unwilling or unable to return for follow-up blood draw

Table S2: Detailed serological diagnostic results for scrub typhus infection

Serology result	Details	% Positivity
IgM and IgG positive for both acute and convalescent phase (n=88)	IgM and IgG titer ≥1:400	27.6% (63)
	IgM titer 4-fold rise and IgG titer ≥1:400	4.8% (11)
	IgM titer ≥1:400 and IgG titer 4-fold rise	1.8% (4)
	IgM and IgG titer 4-fold rise	3.1% (7)
	IgM acute titer ≥1:400 and IgG titer 4-fold rise[a]	1.3% (3)
IgM positive for both acute and convalescent phase (n=7)	IgM titer 4-fold rise	1.8% (4)
	IgM titer ≥1:400	1.3% (3)
IgM positive for both acute and convalescent phase (n=82)	IgG titer 4-fold rise	14.5% (33)
	IgG titer ≥1:400	21.5% (49)
IgM or IgG acute phase positive (n=51)	IgM and IgG acute titer ≥1:400	3.1% (7)
	IgM acute titer ≥1:400 and IgG titer ≥1:400[b]	6.6% (15)
	IgM acute titer ≥1:400[c]	3.1% (7)
	IgG acute titer ≥1:400[c]	9.6% (22)

"a" indicates cases where only the IgM titer in acute phase samples was ≥1:400, with a subsequent 4-fold rise in IgG titers between acute and convalescent phases; "b" indicates cases where the IgM titer in the acute phase was ≥1:400 and IgG titers in both the acute and convalescent phases were ≥1:400; "c" indicates cases where either the IgM or IgG titers in the acute phase alone were ≥1:400
